# Toward the holistic, reference, and extendable atlas of the human brain, head, and neck

**DOI:** 10.1007/s40708-015-0012-4

**Published:** 2015-02-27

**Authors:** Wieslaw L. Nowinski

**Affiliations:** Department of Radiology, University of Washington, Seattle, WA USA

**Keywords:** Brain atlas, Head and neck atlas, Taxonomy

## Abstract

Despite numerous efforts, a fairly complete (holistic) anatomical model of the whole, normal, adult human brain, which is required as the reference in brain studies and clinical applications, has not yet been constructed. Our ultimate objective is to build this kind of atlas from advanced in vivo imaging. This work presents the taxonomy of our currently developed brain atlases and addresses the design, content, functionality, and current results in the holistic atlas development as well as atlas usefulness and future directions. We have developed to date 35 commercial brain atlases (along with numerous research prototypes), licensed to 63 companies and institutions, and made available to medical societies, organizations, medical schools, and individuals. These atlases have been applied in education, research, and clinical applications. Hundreds of thousands of patients have been treated by using our atlases. Based on this experience, the first version of the holistic and reference atlas of the brain, head, and neck has been developed and made available. The atlas has been created from multispectral 3 and 7 Tesla and high-resolution CT in vivo scans. It is fully 3D, scalable, interactive, and highly detailed with about 3,000 labeled components. This atlas forms a foundation for the development of a multi-level molecular, cellular, anatomical, physiological, and behavioral brain atlas platform.

**Electronic supplementary material:**

The online version of this article (doi:10.1007/s40708-015-0012-4) contains supplementary material, which is available to authorized users.

## Introduction

After *The Decade of the Brain* in the 1990s, the twenty first century is considered the century of the brain. Numerous efforts tackle modeling, mapping, and atlasing of the human brain. We recently witness unprecedented big (in terms of scale and funding) and highly publicized brain projects, such as *The BRAIN Initiative* (*Brain Research through Advancing Innovate Neurotechnologies*) [1], *The Human Brain Project* to build a computer model of the human brain [1, 2], *The Connectome* *Project* to map structural connections [1], *The Big Brain* providing very high-resolution images [3], and *The Allen Brain Atlas* to map gene expression integrated with the underlying neuroanatomy [4, 5].

Despite their novelty, complexity, and brain knowledge expansion, none of these challenging initiatives addresses the basic issue that a fairly complete neuroanatomical model of the whole human brain, which is required as the reference in brain studies and clinical applications, does not exist.

The existing (commercially available) brain atlases, such as *ADAM* [6], *Cerefy* [7], *Digital Anatomist* [8], and *Voxel*-*man* [9], are anatomically incomplete; moreover, besides our *Cerefy* atlases [10], none of these atlases has been used in clinical applications.

In order to capture structural, functional, and molecular variations, numerous endeavors aim at the creation of population-based and spatiotemporal atlases [11–13], including our efforts on the probabilistic functional atlases [10, 14], the population-based stroke atlas [15], and the atlas of cerebrovascular variants [16]. However, these atlases have some limitations [17], such as fuzziness due to averaging, low parcellation, limited anatomical content, and they are often targeted at specific disease types.

Our contribution to these global, brain-related efforts is to create adult human brain atlases and develop atlas-based applications. For over two decades, we have developed 35 brain atlases, licensed to 63 companies and institutions, and made available to medical societies, organizations, medical schools, and individuals. In contrast to the ongoing initiatives, our approach is (1) guided by research, clinical, and market perspectives; (2) top-down; and (3) holistic.

The atlases developed so far by us can be grouped in three categories, namely, derived from print materials, in vivo imaging, and population (including patient-specific) data.

Our ultimate goal is to create a holistic atlas of the whole adult human brain along with the head and neck. The atlas is three-dimensional (3D), advanced, detailed, interactive, accurate, reference, realistic, high resolution, fully parcellated, completely labeled, spatially consistent, stereotactic, user friendly, extendable (scalable), composable, dissectible, explorable, and modular.

This work aims to present the taxonomy of the currently developed atlases and to address the design, content, functionality, and current results in the holistic atlas development as well as atlas usefulness and future directions.

## Materials and methods

### Materials

A normal, adult, male specimen (W.L.N.) has been scanned over many years on various scanners and with variable pulse sequences. A single specimen was employed to ensure spatial consistency and extendibility of the atlas. In order to build the brain model (along with the skin, glands and head muscles), multispectral magnetic resonance (MR) data were acquired on 1.5 Tesla (T), 3 T/8 channels, 3 T/32 channels, and 7 T instruments, and with T1-weighted (T1W), T2-weighted (T2W), magnetization prepared rapid gradient echo (MP-RAGE), 2D time-of-flight (TOF), 3D TOF, susceptibility-weighted imaging (SWI), spoiled gradient-recalled (SPGR), and diffusion tensor imaging (DTI) pulse sequences. The skull model was created from a high-resolution computed tomography (CT) scan. To create the neck vessels, MR data were acquired on a 3 T scanner with phase contrast angiography (PCA) and M2D (Philips term) inflow pulse sequences.

### Atlas taxonomy

**Fig. 1:**
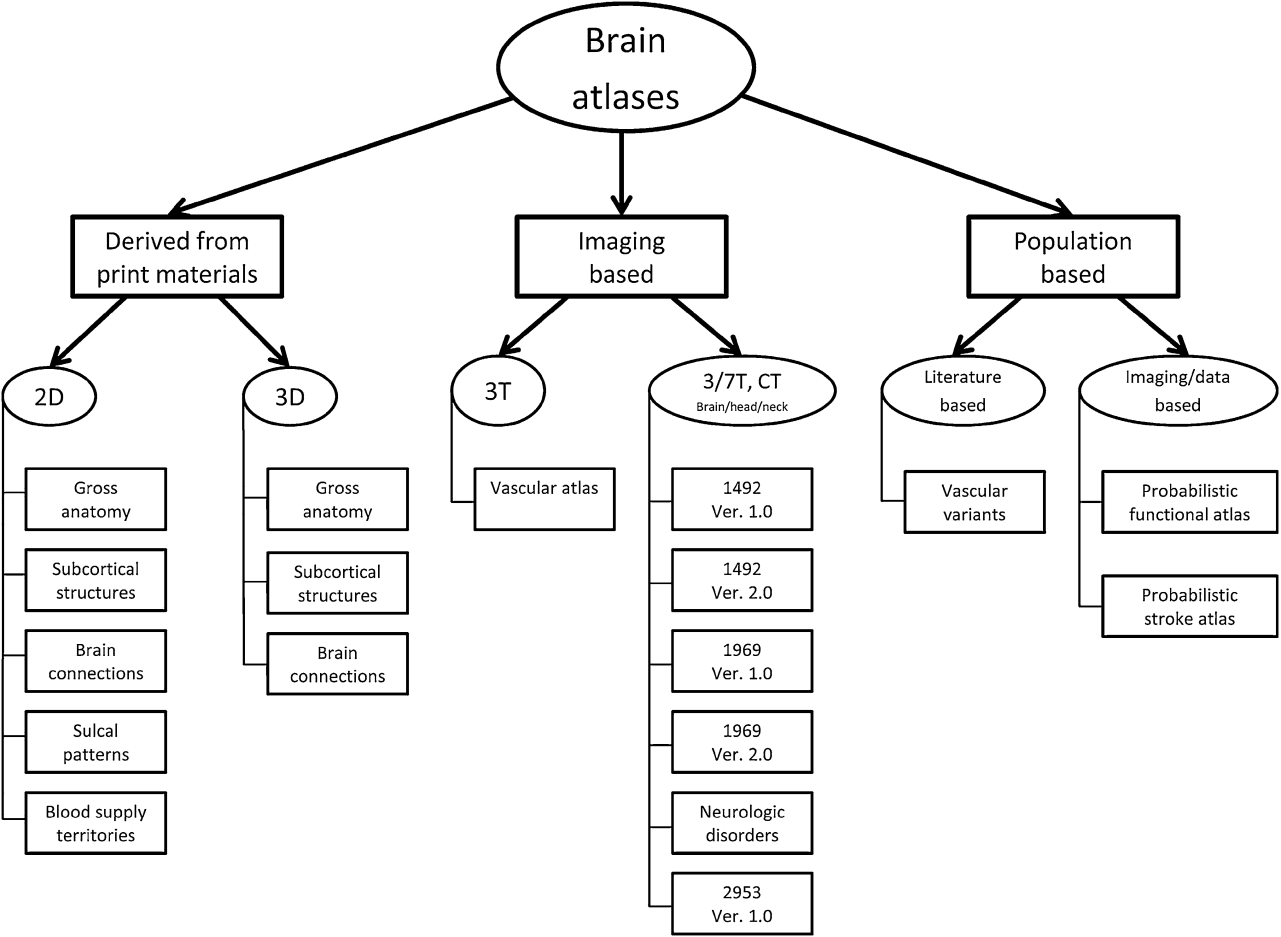
Taxonomy of the currently developed brain atlases

Several taxonomies of brain atlases have been proposed, and we also have introduced some earlier [14, 18]. Here, we present and discuss the taxonomy of all the brain atlases created by us. This taxonomy is diagrammed in Fig. 1.

We have developed three major families of atlases derived from print materials, in vivo imaging, and population (including patient-specific) data. The print material-based family has been successful, several atlases have been developed [7, 19–22], and brain atlas libraries incorporated into surgical workstations [10]. However, despite integration of multiple atlases with complementary contents (gross anatomy [23], deep structures [24], brain connections [25], and sulcal patterns [26]) within a single system with their enhanced electronic versions [27] as well as extending these atlases to 3D [27, 28], an accurate match of the component atlases and content extension were not feasible. This was the main reason to start constructing a new generation of brain atlases, based on imaging of a single specimen. Another reason is that none of the recent big brain projects addresses a construction of a complete neuroanatomical model. Such a reference model is required in brain studies and clinical applications as well as in big projects, such as *BRAIN* *Initiative* and *The Human Brain Project* (see the Sect. 4).

Our endeavor shifts the brain atlas development paradigm FROM fragmented studies and mosaic content, “copy and paste” neuroanatomy, static presentation, and tedious neurological description TO holistic representation, truly 3D structural, vascular, connectional and functional neuroanatomy, dynamic brain de/composition (from blocks to brain), dysfunction brain atlases, and brain at work. Several atlases have been created within this family (an atlas of cerebral vasculature [29] as well as the human brain in 1492 [30, 31] (two editions), 1969 [32, 33] (2 editions) along with mobile versions on iPad [34] and Android-based [35], and 2953 [36] pieces along with the 3D atlas of neurologic disorder (on desktop [37] and iPad [38])), and they excellently illustrate our strategy and paradigm shift.

Several atlases have been created for neurologic disorders [39–41]. They provide disorder—localization relationships and tools for exploration as well as correlate cerebral pathology with the underlying neuroanatomy and the resulting neurological deficits. These atlases present various simulated cerebral pathologic situations (lesions) in 3D that are labeled with the resulting disorders along with the surrounding highly parcellated neuroanatomy. Disorders are linked to textbook materials and described in terms of the resulting signs, symptoms and/or syndromes (for instance in the *Stroke Atlas* [41], the disorders are compiled for ischemic stroke, hemorrhagic stroke, and cerebral aneurysms).

The current reference atlas is by definition deterministic, where the brain can be easily composed and decomposed, and each cerebral component is uniquely labeled, freely manipulated, and presented within its user-selected surrounding context. These operations are not feasible in existing population-based atlases, which are typically mosaic type.

As a single-specimen atlas does not contain variability, the third family of atlases has been created to deal with this problem. Our current efforts to create the holistic, deterministic (a single specimen), and reference atlas have taken more than a decade and this is still work in progress. In our opinion, the development of the holistic, probabilistic, and reference atlas is an enormous and expensive task. Therefore, our concept of handling variability is to integrate it around the reference deterministic atlas. A good illustration of this approach is our atlas of arterial cerebral variants (see the Sect. 4). The third family of atlases is based on imaging and non-imaging population data. We have used patient-specific data to develop atlases for clinical applications. The atlases developed so far in this group (along with new patented or patent-pending solutions) are exploratory to illustrate anatomic [16], functional [10, 14, 42, 43], and pathologic [15] variability.

### Atlas design principles

Ten principles of atlas design are the following

1. *Electronic* The atlas is electronic (digital) to make its presentation, manipulation, enhancement, extension, automatic processing, and distribution easy.
2. *3D* The atlas is 3D and of high spatial resolution to avoid spatial inconsistency and sparseness. It provides both surface and sectional anatomy.
3. *In vivo* The atlas is created from in vivo advanced, multispectral MR, and high-resolution CT imaging to avoid post-mortem artifacts and spatial distortions resulting from specimen processing, particularly, histological sectioning.
4. *Holistic/single specimen* We aim to build a maximally complete model of the whole brain along with the head and neck (an anatomically holistic approach), in contrast to just any part or component of it (a mosaic approach). Any new component or (sub)system added to the existing model must precisely fit to it, which is facilitated (but not obviously feasible due to geometric imaging distortions and artifacts) by the use of a single, reference, rescannable brain specimen.
5. *Top*-*down design* The complexity of the cerebral model has been continuously increasing by using a top-down approach. The whole brain has been continuously subdivided into smaller and smaller components, and extended with more and more (sub)systems, and the head and neck have been included. Consequently, several versions of the atlas are available now, and new extended, versions will be progressively upcoming.
6. *Deterministic/labelable model* The boundaries between the neighboring components shall be uniquely determined to avoid (or minimized) neither holes nor overlaps between them and to make any location (on the 3D model and the MR scan) uniquely labelable (being named) or characterized with various features. Hence, these brain, head, and neck components form 3D “blocks”.
7. *Scalable design/from blocks to brain, head, and neck* Both the model and the atlas user interface are scalable. The complete model, or any of its parts, is easily composed (assembled) from or decomposed (disassembled) into blocks at multiple levels with a few clicks of the mouse. The user interface is extendable to handle new modules.
8. *Stereotactic reference* The model is placed in a stereotactic (bicommissural) coordinate system and forms a stereotactic reference with coordinate readout.
9. *Affordable/easy to use* The atlas runs on a standard computer (PC and MAC) as well as on mobile platforms (iPad and Android-based). It is easy to operate, and any system/configuration can be created just with a few clicks.
10. *Beautiful brain, head, and neck* The atlas is designed with an emphasis on esthetics and, particularly, model coloring to make the brain, head, and neck as well as the user interface beautiful.

### Atlas content

At present (i.e., included into the recent version [36]), the atlas of the brain, head, and neck contains about 3,000 following components:

- *brain* divided into the left and right hemispheres, cerebrum, cerebellum, and brainstem;
- *cerebellum* divided into the left and right hemispheres;
- *brainstem* divided into the left and right parts, and parcellated into the midbrain, pons, and medulla;
- cerebral cortex completely parcellated into lobes, gyri, and gyri with sulci as well as 3D Brodmann’s areas (not integrated yet with [36]);
- *spinal* *cord* divided into the left and right parts;
- *white matter* parcellated into cerebral, posterior fossa, and deep white matter;
- *gray matter nuclei* completely parcellated;
- *ventricular system* completely parcellated;
- *intracranial vasculature* (arteries, veins, and dural sinuses) parcellated into more than 1,300 vessels, the smallest of 0.08 mm in diameter;
- *intracranial arteries* grouped into the internal carotid, anterior cerebral, middle cerebral, posterior cerebral, vertebral and basilar arteries, and the circle of Willis, each group completely parcellated;
- *intracranial veins* grouped into superficial, deep, and posterior fossa veins, each group completely parcellated;
- *dural sinuses* completely parcellated;
- *extracranial vasculature* grouped into arteries and veins, each group completely parcellated;
- *white matter tracts* grouped into associations, commissures, projections, and posterior fossa tracts, each group completely parcellated;
- *cranial nerves* (CN) grouped into CN I–CN XII, each group completely parcellated, along with the nuclei with more than 630 components;
- *head muscles* grouped into extra-ocular, facial, masticatory, and other muscles, each group completely parcellated;
- *glands* grouped into mouth and other glands, each group completely parcellated;
- *skull* completely parcellated into all 29 bones;
- *skin* divided into the left and right parts;
- functional (*auditory* and *visual*) systems parcellated;
- *cervical spine* parcellated into vertebrae;
- *pharynx* parcellated (not integrated yet with [36]);
- *teeth* completely parcellated into all 32 teeth (not integrated yet with [36]).

This content is grouped in our recent atlas [36] into 17 modules (tissue classes): central nervous system, deep structures, ventricles, white matter, white matter tracts, intracranial arterial system, intracranial venous system, head muscles, glands, extracranial arteries, extracranial veins, skull, skin, neck, visual system, and auditory system.

### Atlas functionality

The atlas provides a rich functionality for model manipulation and exploration. These functions are grouped into seven main clusters for.

1. *Structure selection* Select and/or deselect all, (tissue) classes clusters, classes, groups, individual structures, and/or left, right or both sides (during 3D scene compositing and/or decompositing operations). These operations are supported by two-way mapping between the 3D view (where the 3D model is displayed) and the anatomical index.
2. *3D model real*-*time manipulation* The composed 3D scene can be displayed in 3D and manipulated in real time by performing its rotation (arbitrarily or around any reference axis), zooming, and panning; setting predefined views (anterior, posterior, superior, inferior, left and right); and starting/stopping animation (3D scene auto rotation).
3. *Virtual dissections* The 3D model is dissectible by means of 3D cutting of the cerebrum, cerebellum, brainstem, spinal cord, white matter, and skull. Dissections are 3D by means of seven cutting planes: two planes (to cut the model from its both ends) per axial, coronal and sagittal orientations and an arbitrary plane in the viewing direction. This operation allows exposing structures lying inside (to see through the brain) as well as enables a simultaneous display of surface and sectional anatomy in any orientation.
4. *Scan manipulation* The main structural (MP-RAGE) scan can be shown along with the 3D model, and its axial, coronal, and sagittal planes scrolled and manipulated while being displayed in 3D.
5. *Atlas content querying* Similarly to a two-way (3D view––anatomical index) mapping, querying is also done in a two-way. Either the 3D scene or the index can be queried. When querying a structure in the 3D scene about its properties, its name is highlighted is the index and a label is shown in the 3D view with the name (and the diameter for the vessels). The labels can be placed permanently and manipulated in 3D. In general, a vector of items can be provided, such as literature materials or references, like in [29, 37] atlases. When querying the index about structure location, the structure (or a group of structures) is (are) highlighted in the 3D view.
6. *Coordinates and distance readout* As the atlas is stereotactic, 3D coordinates of any location are provided and a distance between any two atlas points can be measured.
7. *Supporting functions* They provide image saving, clearing labels and highlights, and getting information and help.

## Results

To date, we have developed 35 brain atlases for education [20], [44, 45], research [18, 46] and clinical applications [47], mainly in neuroradiology [28, 48, 49] neurosurgery [10, 50, 51], and neurology [39–41]. Dedicated atlas-based solutions have been proposed and developed for stereotactic and functional neurosurgery [10, 19, 42, 43, 50, 52–54], stroke image analysis and prediction [15, 55], scan interpretation [45], brain cancer [49], psychiatry (schizophrenia) [56], and human brain mapping [21, 46, 51]. We have extensively published these efforts in about 200 publications, including journal and conference papers as well as clinical abstracts.

Our atlases have been (1) licensed to 63 companies and institutions globally; (2) installed in over 1,500 surgical workstations by major surgical companies (including Medtronic/USA, Brainlab/Germany and Elekta/Sweden); (3) distributed to medical schools and individual clinicians by a leading medical publisher Thieme (over 7,500 copies of 15 atlases, some of them bestsellers); (4) made available publicly to numerous medical societies (e.g., World Stroke Organization, Society for Brain Mapping and Therapeutics), organizations, and individuals; (5) globally recognized receiving 23 awards, where 20 awards are from leading medical societies, including top ones (*Summa cum laude* in 1997, 2008, 2012, and 2014 from American Society of Neuroradiology and *Magna cum Laude* (considered a Radiological Oscar) with *Excellence in Design* in 2009 from Radiological Society of North America); and (6) featured on the *Discovery Channel, CNN,* and in *The Wall Street Journal*. We have introduced electronic brain atlases to stereotactic and functional neurosurgery [10, 54], mainly for deep brain stimulation to treat Parkinson’s disease and other disorders. Hundreds of thousands of patients have been treated by employing our atlases.

The holistic and reference atlas constructed to date contains the brain, head, and neck with about 3,000 components and is made available [36]. The virtual model in the atlas contains structure [17], intracranial vasculature [57], white matter tracts [58], cranial nerves with nuclei [59], head muscles and glands [60], extracranial vasculature [61], and a complete skull added recently reconstructed from a high-resolution CT scan [62], each validated against the state-of-the-art literature.

**Fig. 2:**
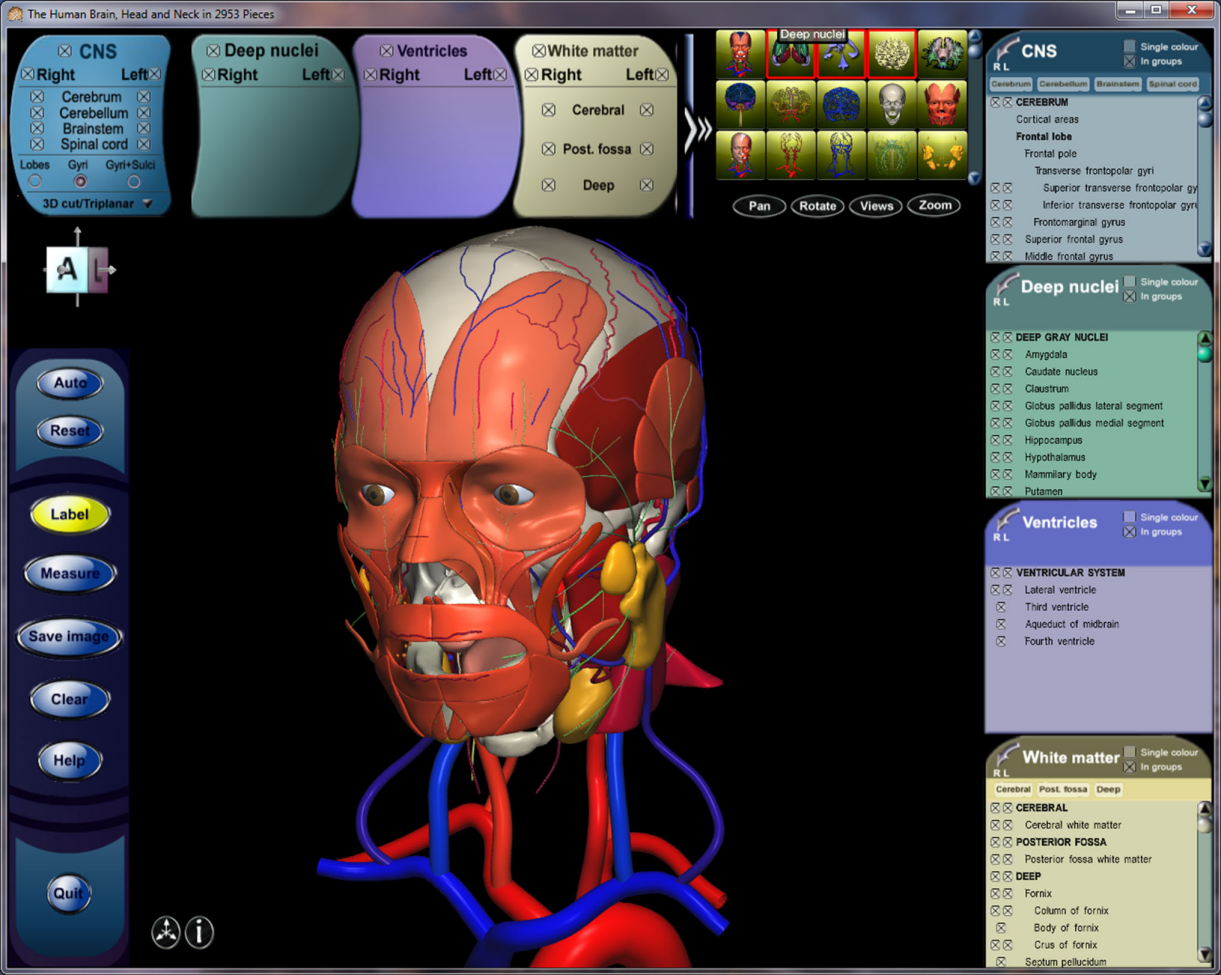
User interface of the 2953 atlas: controls, anatomical index, and 3D view with the brain, head, and neck model

**Fig. 3:**
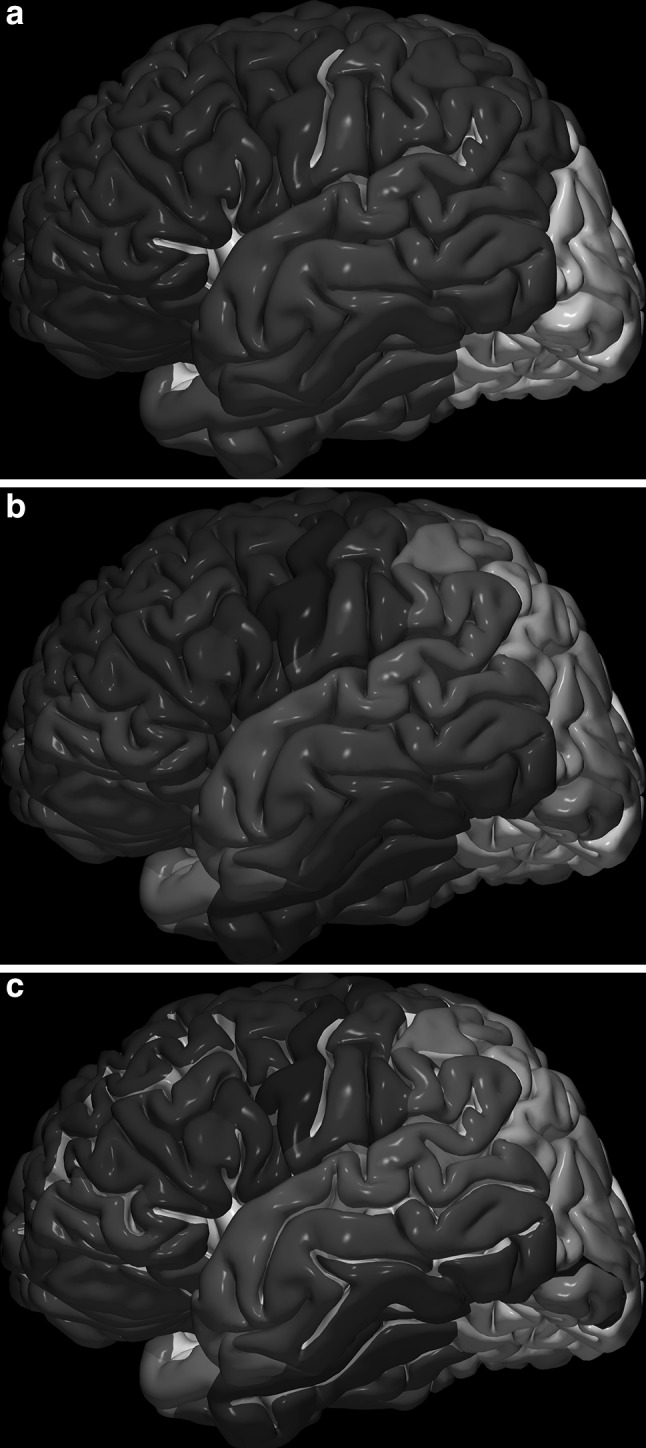
Cortical parcellation: **a** lobes; **b** gyri; **c** gyri and sulci

**Fig. 4:**
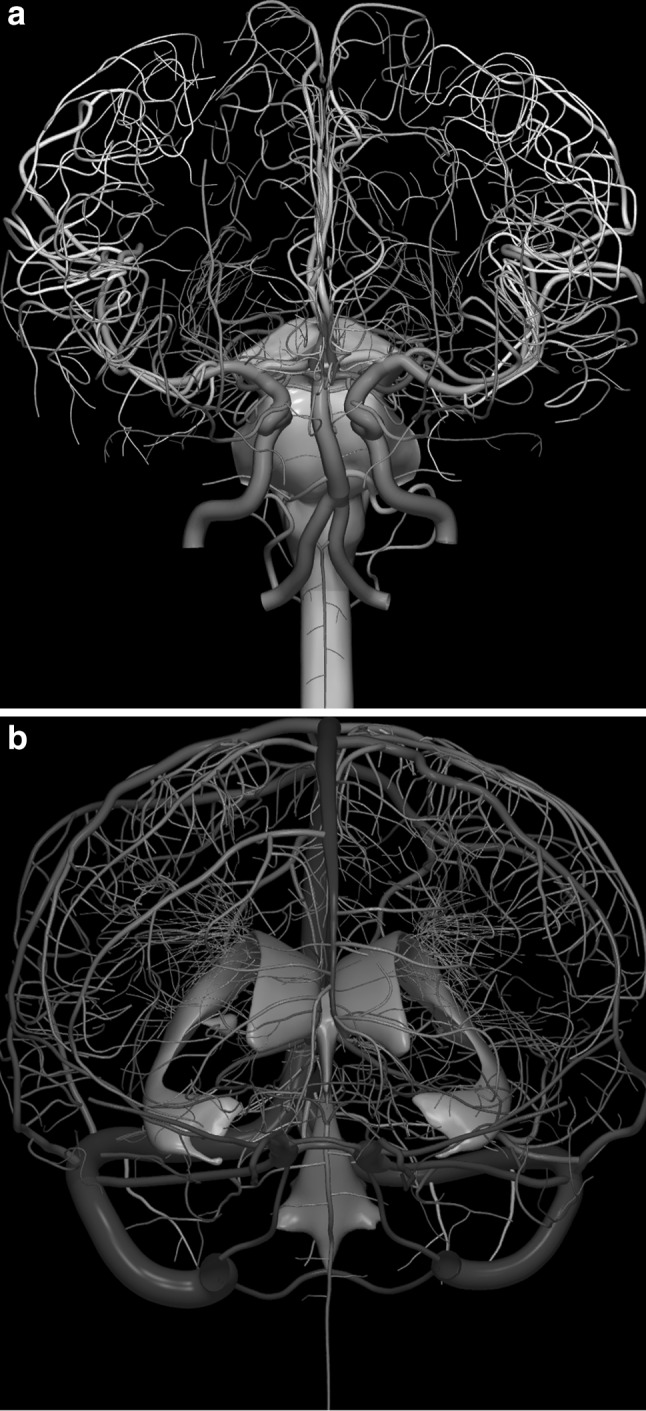
Cerebrovasculature: **a** arterial system (along with the brainstem and spinal cord); **b** venous system (along with the ventricles)

**Fig. 5:**
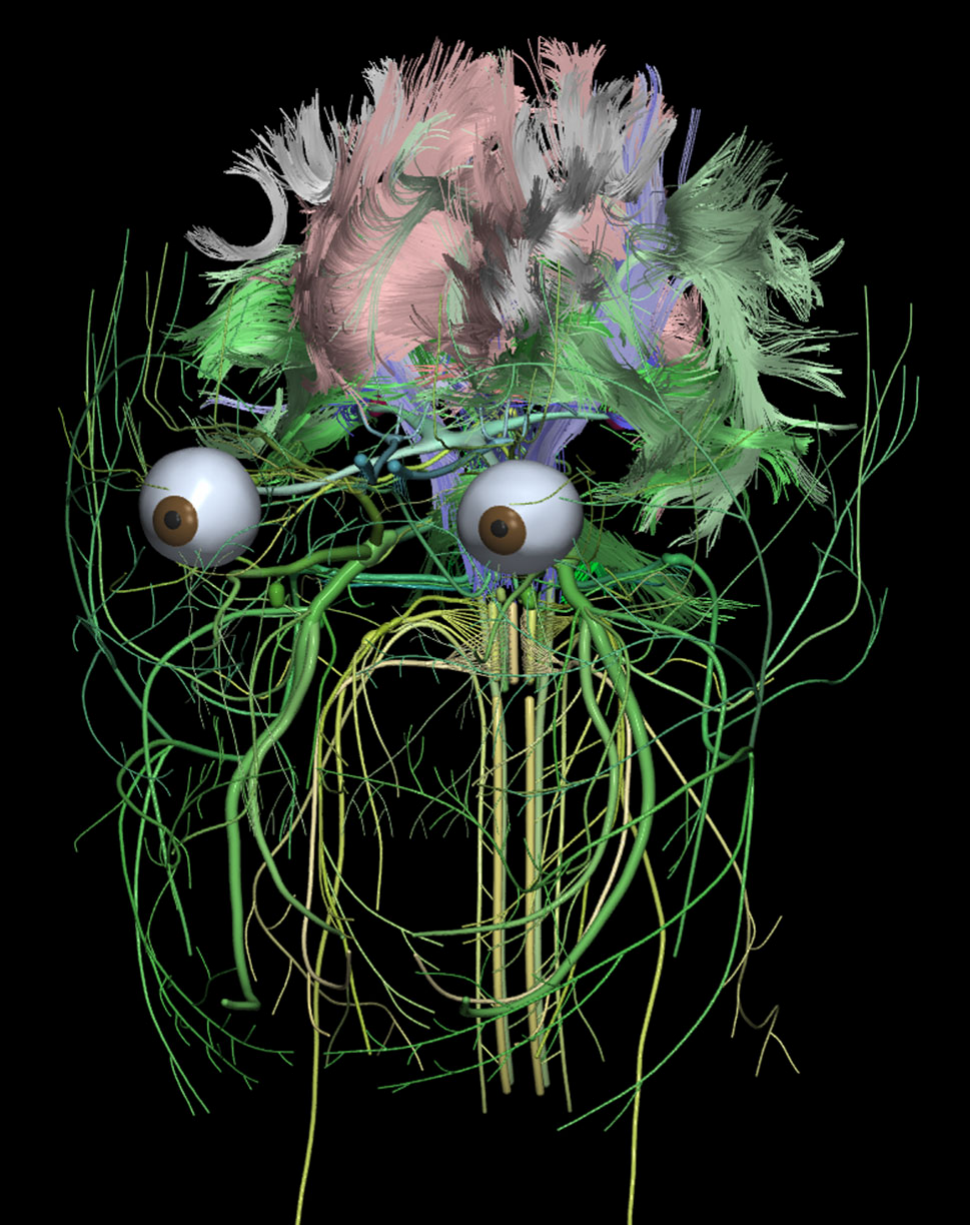
Cranial nerves along with white matter tracts and visual system

**Fig. 6:**
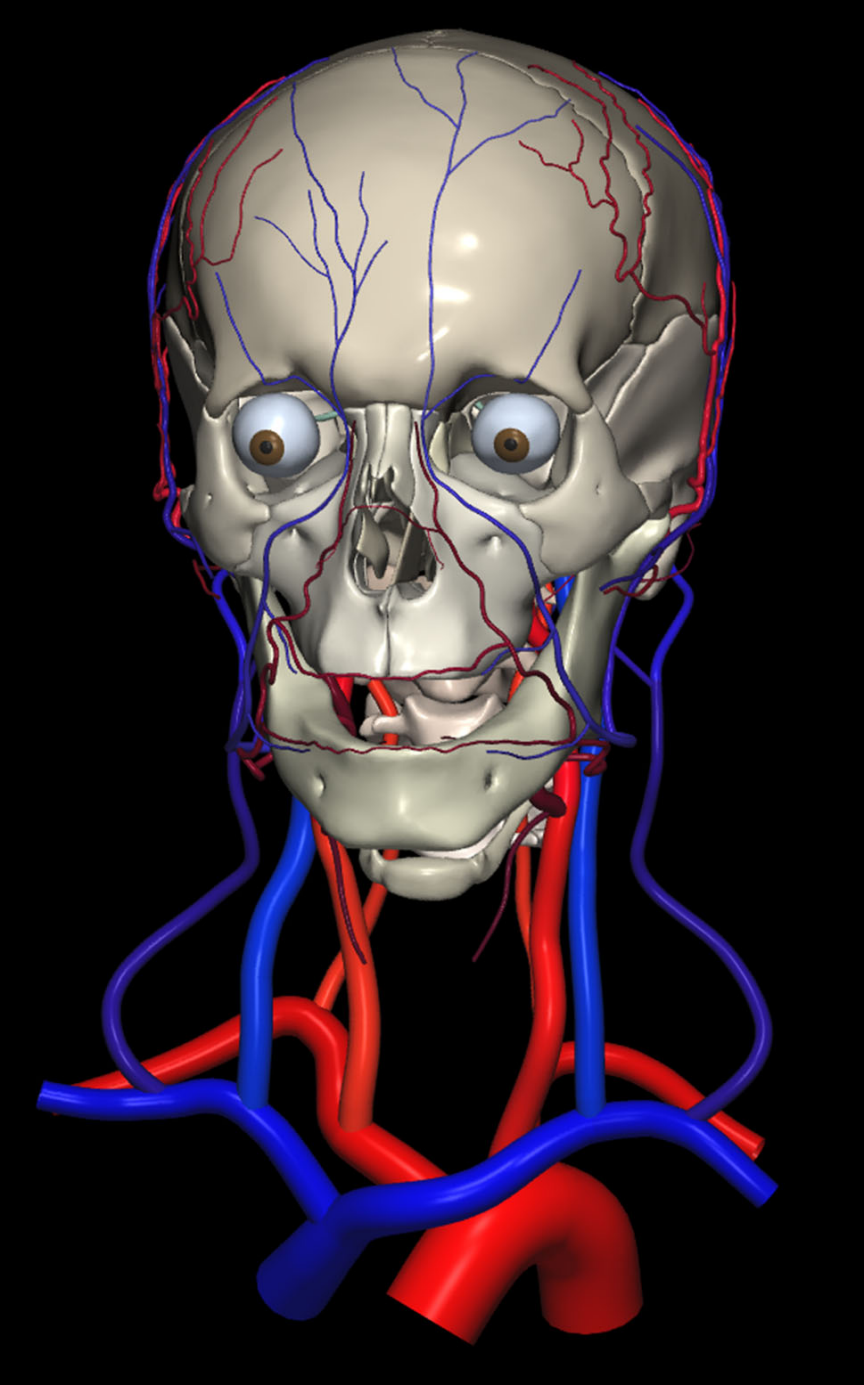
Skull, cervical spine, extracranial arteries, extracranial veins, and visual system

**Fig. 7:**
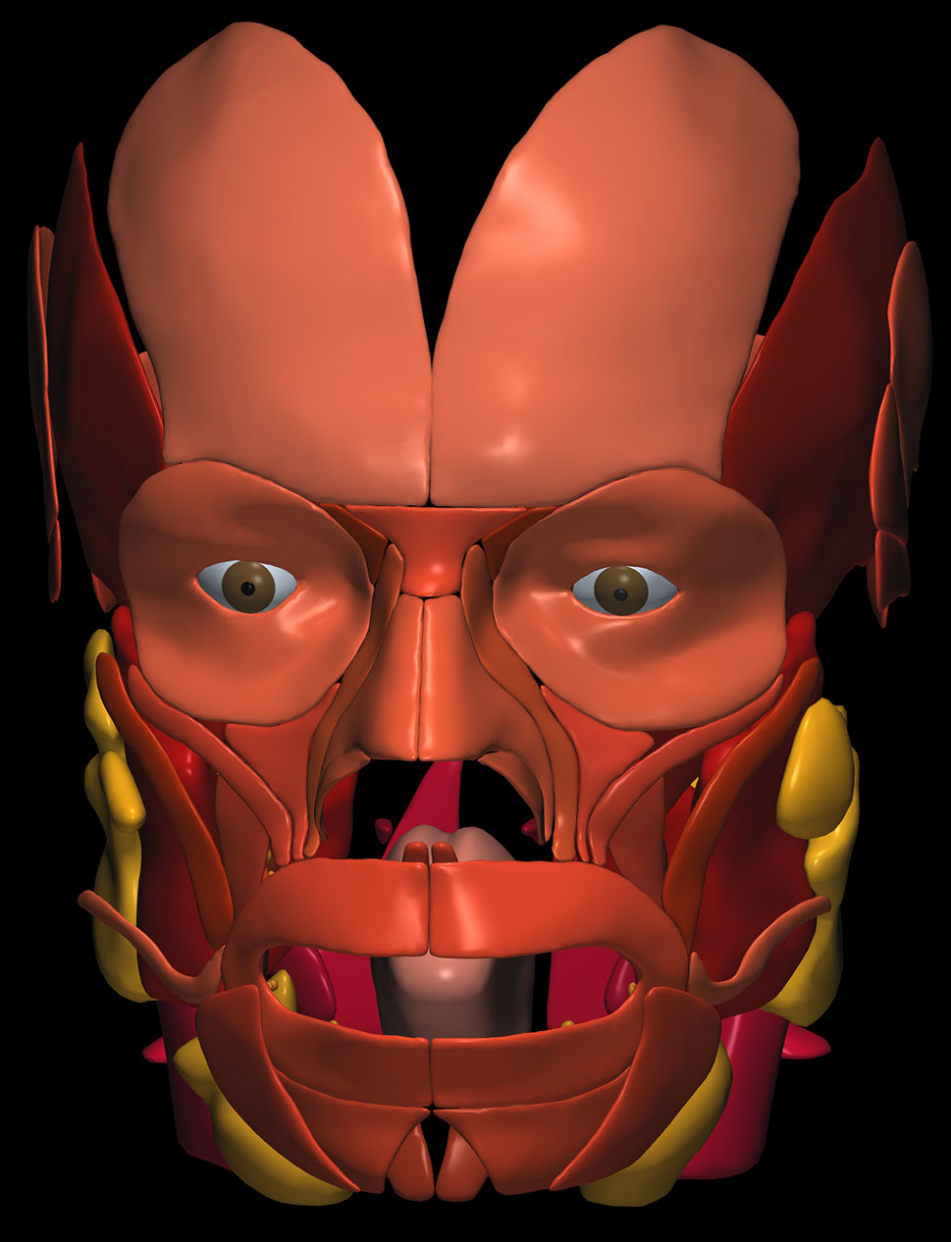
Head muscles, glands, and visual system

**Fig. 8:**
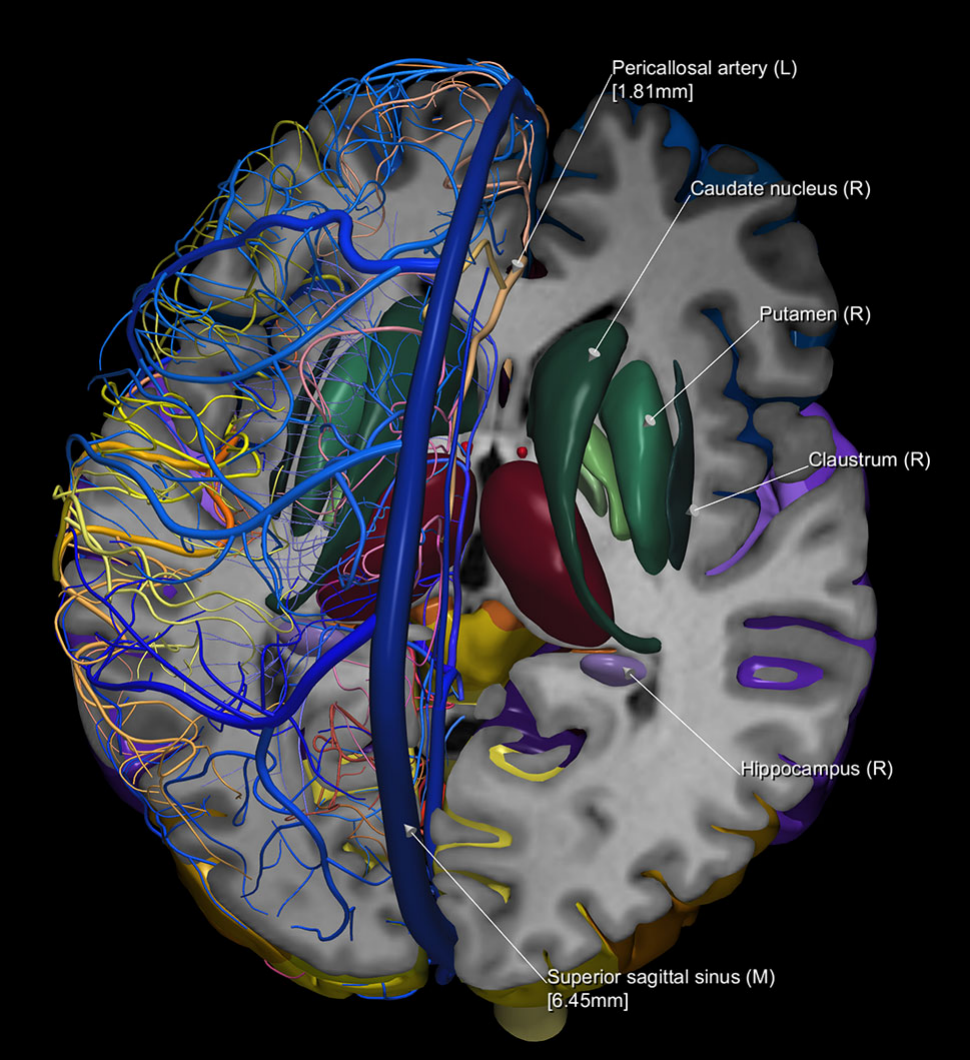
Illustration of brain cutting and labeling

The images below illustrate the currently developed atlas: user interface (Fig. 2), cortical parcellation (Fig. 3), cerebrovasculature (Fig. 4), cranial nerves with white matter tracts (Fig. 5), skull, cervical spine, and extracranial vessels (Fig. 6), head muscles and glands (Fig. 7), and brain cutting and labeling (Fig. 8).

## Discussion

Our atlas work is impactful despite being of a small funding scale. We have developed 35 commercial atlases and additionally numerous research prototypes presented at clinical and educational meetings. These brain atlases have a wide range of use. We have already developed atlas-based applications ranging from education to research to clinical use.

### Advantages

Our approach to brain atlas creation has several advantages. It leads to a construction of an anatomically holistic brain model, much more complete and detailed than any existing anatomical brain models created so far (to our best knowledge). Building it from in vivo data eliminates post-mortem artifacts and spatial distortions resulting from histological sectioning. The use of a single, rescannable (over long time) specimen allows us to build a spatially consistent model, easily expandable with new scans, benefiting this way of fast developments in diagnostic imaging. A modular design provides an expandable content and scalable user interface. Virtual brain dissection enables the user to see, e.g., cortical areas hidden in the folded cortex, vessels cursing within the sulci, and deep structures with the surrounding parenchyma. 3D labeling enables the user to interactively explore the labeled cerebral model or any part of it with the labels attached to the components while being manipulated. The compositing and decompositing mechanism (from blocks to brain) allows the user to build any region of interest or (sub)system as well as to present local anatomy within its global context with a few clicks of mouse. The labelable triplanar display (especially useful for neuroradiology residents and neuroradiologists) merges sectional with surface anatomy. Mapping of the atlas to patient’s data can be done using standard techniques for MR-to-MR registration. Then, the deformation field calculated for the structural scan of the brain specimen shall be applied to the corresponding 3D cerebral model. We have designed the atlases to run on a standard computer and mobile platforms (including iPad [34] and Android-based [35]) in order to make them affordable to a wide spectrum of users ranging from a layman to researcher to medical student to clinician. By extending the model to the head and neck, the applicability of the atlas is expanded.

The atlases of neurologic disorders, combining neuroanatomy, neuroradiology, and neurology, are supportive in a recent trend in radiology (from volume- to value-based) aiming to enhance neuroradiologist-clinicians and neuroradiologist-patient communication. For instance, communicating a stroke situation is complicated and time consuming in a time critical condition and new tools are needed for this purpose. Then, the *Stroke Atlas* [41] may serve as an aid in patient-doctor communication facilitating to explain the situation to the patient and/or his or her family. Moreover, as this stroke atlas is self-explanatory and easy to use, it enables a layman to get familiarized with normal neuroanatomy and understand what happens in stroke.

### Deterministic versus population-based atlases

Population-based atlases are particularly useful in anatomical interpretation of functional imaging observations (especially to highlight the uncertainty regions), studying intersubject variability and left–right asymmetries as well as they can distinguish abnormalities from normal variants, and detect group-specific features and average patterns. Moreover, dynamic atlases with time-varying data help analyze the growth rate and lifelong normal changes [13].

As both deterministic and population-based atlases have their own merits, their combination is advantageous as illustrated, for instance, by us in [52], where a highly anatomically parcellated atlas of the deep structures is integrated with a low parcellation, high spatial resolution, and population-based atlas of the subthalamic nucleus applicable for deep brain stimulation. Then, the population-based atlas facilitates to define the target (the point with the highest probability), while the anatomical atlas (containing target’s anatomical neighborhood) the trajectory to it.

The averaging process, typically applied to construct population-based atlases, washes away the actual anatomical information. Consider, for instance, arterial variants of the circle of Willis (CoW). Averaging of the variants of an incomplete CoW in 3D results in a complete averaged CoW with lost information on variants’ variability. To avoid this problem in a population-based atlas, we have proposed to include all the variants and present individually each of them in the context of a single reference deterministic atlas [16]. These examples illustrate that a detailed anatomical atlas is a critical component, independent of and not replaceable by a population-based atlas.

### Limitations

Most of the current limitations are probably because this work is in progress. Our initial focus has been to develop an accurate and detailed anatomic model. This is because half of knowledge in several disciplines is neuroanatomy, including neurosurgery, neurology, neuroradiology, and neuroscience. Moreover, neuroanatomy and neuroscience are reported most difficult in medical education. With the future developments, as discussed below, the power and usefulness of the reference atlas will be growing.

### Future developments

As stated by Dr. Anne Osborn in the foreword to the 1492 atlas [31]: “*you hold the future in your hands*” and “*applications of this tool are exciting to contemplate.*” Our design facilitates construction of a family of various atlases, such as application-specific, patient-oriented, layman-oriented, and medical speciality-oriented. This modular and scalable atlas can easily be extended by acquiring new scans of the same specimen, integrating new studies and simulations mapped to the same stereotactic atlas space, and by synthesizing small components which are beyond the current imaging resolution. Therefore, we keep brain scanning, segmenting, modeling, editing, cerebral model cutting, and structure synthesizing. We plan to integrate imaging (including structural, functional, connectional, and molecular images) and non-imaging data, literature materials, and simulation results, thereby growing the current atlas both “up” and “down.” This effort may take decades to create a multi-level molecular, sub-cellular, cellular, circuital, anatomical, physiological, and behavioral brain atlas platform (provided that funding is available).

The 3D atlas of neurologic disorders [37] can easily be extended by adding many more synthesized lesions and linking them with clinical cases. This may expand the applicability of the atlas to become an aid for neurological diagnosis and pathology localization, particularly, in the radiology deprived regions, where diagnostic imaging is neither available nor affordable

### Usefulness in big brain projects

This atlas is potentially useful in the recent big brain projects, including the *BRAIN Initiative* and *The Human Brain Project*. It can serve as a (1) reference for enormous amounts of data that will be generated; (2) framework for result integration and interpretation; (3) vehicle to present and disseminate the discoveries from science (and also within scientific disciplines) to medicine to public; (4) potential “Wikipedia” for the brain community; and (5) tool reducing a difficulty barrier (as the brain model is made easy and beautiful) in order to train a new wave of neurotechnologists and neuroscientists, make neurologic disorders more understandable, and educate public.

### Summary

We have developed to date 35 commercial brain atlases (along with numerous research prototypes), licensed to 63 companies and institutions, and made available to medical societies, organizations, medical schools, and individuals. These atlases have been applied in education, research, and clinical applications, mainly in neuroradiology, neurosurgery, and neurology. Dedicated atlas-based solutions have been proposed and developed for stereotactic and functional neurosurgery, stroke image analysis, scan interpretation, brain cancer, psychiatry (schizophrenia), and human brain mapping. Hundreds of thousands of patients have been treated by using our atlases.

Our ultimate objective is to create a holistic, reference, and extendable atlas of the whole adult human brain along with the head and neck. The first version of such an atlas has been developed and made available. The atlas has been created from multispectral 3 and 7 T and high-resolution CT in vivo scans. It is fully 3D, scalable, interactive, and highly detailed with about 3,000 labeled components.

This atlas forms a foundation for the development of a multi-level molecular, cellular, anatomical, physiological, and behavioral brain atlas platform.

## Electronic supplementary material

Below is the link to the electronic supplementary material.

Supplementary material 1 (DOCX 18 kb)
