# Supplementary material for: Toward the holistic, reference, and extendable atlas of the human brain, head, and neck
Source: Brain Inform. 2015 Feb 27;2(2):65–76. doi: 10.1007/s40708-015-0012-4 (PMC4883147; doi:10.1007/s40708-015-0012-4)
Supplement: Supplementary file 1 — Supplementary material 1 (DOCX 18 kb) [file 40708_2015_12_MOESM1_ESM.docx]

Prof. Wieslaw L. Nowinski, DSc, PhD is affiliate professor at the Department of Radiology, University of Washington, Seattle, WA. His research includes brain atlases, stroke, deep brain stimulation, brain quantification, neuroinformatics, medical image processing, virtual reality, computer-assisted diagnosis and treatment, and future directions in computer-aided radiology and surgery.

He has 543 publications, filed 51 patent applications (32 already granted, 15 in US and 8 Europe (EP)), and developed with his team 35 brain atlas products used worldwide in neurosurgery, neuroradiology, neurology, brain mapping, and neuroeducation. These atlases have been licensed to 63 companies and institutions. Fifteen brain atlases have been distributed by Thieme Medical Publishers, New York - Stuttgart. The atlases are also installed in 1,500 surgical workstations.

Dr. Nowinski has been conferred with 42 awards and honours; 25 awards are from leading medical societies, including *Pioneer in Medicine* in 2013 from Society for Brain Mapping & Therapeutics, *Magna cum Laude* (radiological Oscar) from Radiological Society of North America in 2009 and 2004; *Magna cum Laude* from European Congress of Radiology in 2000; *Summa cum Laude* in 2014, 2012, 2008 and 1997, and *Magna cum Laude* in 2009 and 2005 from American Society of Neuroradiology. He has been an *Asian Innovation Awards* 2010 finalist (for his stroke work) and in top 3 nominees for *European Inventor Award* 2014 organized by the European Patent Office in category of *lifetime achievement* (for his brain atlas work). Dr. Nowinski has been named *The Outstanding Pole* (in the world) in 2012. His work has been featured in *The Wall Street Journal* and on *The Discovery Channel, CNN and Channel News Asia,* among others.

[www.WieslawNowinski.com](http://www.WieslawNowinski.com)
